# RNA interference of trehalose-6-phosphate synthase and trehalase genes regulates chitin metabolism in two color morphs of *Acyrthosiphon pisum* Harris

**DOI:** 10.1038/s41598-020-80277-2

**Published:** 2021-01-13

**Authors:** Guang Wang, Yuping Gou, Sufan Guo, Jing-Jiang Zhou, Changzhong Liu

**Affiliations:** 1 College of Plant Protection, Gansu Agricultural University, Lanzhou, 730070 Gansu China; 2 Biocontrol Engineering Laboratory of Crop Diseases and Pests of Gansu Province, Lanzhou, 730070 Gansu China

**Keywords:** Biological techniques, Developmental biology, Molecular biology

## Abstract

Trehalose-6-phosphate synthase (TPS) and trehalase (TRE) directly regulate trehalose metabolism and indirectly regulate chitin metabolism in insects. Real-time quantitative PCR (RT-qPCR) and RNA interference (RNAi) were used to detect the expressions and functions of the ApTPS and ApTRE genes. Abnormal phenotypes were found after RNAi of *ApTRE* in the *Acyrthosiphon pisum*. The molting deformities were observed in two color morphs, while wing deformities were only observed in the red morphs. The RNAi of *ApTPS* significantly down-regulated the expression of chitin metabolism-related genes, UDP-N-acetyglucosamine pyrophosphorylase (*ApUAP*), chitin synthase 2 (*Apchs-2*), Chitinase 2, 5 (*ApCht2*, *5*), endo-beta-N-acetylglucosaminidase (*ApENGase*) and chitin deacetylase (*ApCDA*) genes at 24 h and 48 h; The RNAi of *ApTRE* significantly down-regulated the expression of *ApUAP*, *ApCht1*, *2*, *8* and *ApCDA* at 24 h and 48 h, and up-regulated the expression of glucose-6-phosphate isomerase (*ApGPI*) and Knickkopf protein (*ApKNK*) genes at 48 h. The RNAi of *ApTRE* and *ApTPS* not only altered the expression of chitin metabolism-related genes but also decreased the content of chitin. These results demonstrated that *ApTPS* and *ApTRE* can regulate the chitin metabolism, deepen our understanding of the biological functions, and provide a foundation for better understanding the molecular mechanism of insect metamorphosis.

## Introduction

Chitin is a polymer of N-acety1-β-d-glucosamine and also a major component of the insect cuticle. It is vital to the peritrophic matrix, which acts as a permeability barrier between food and midgut epithelium, promotes digestion and protects the brush border from mechanical disruption^1,2^. The biosynthesis and degradation of chitin are influenced by enzymes, food substrates, energy suppliers and intracellular environment^3^. Rate-limiting enzymes, glutamine-fructose-6-phosphate aminotransferase (GFAT), UDP-N-acetyglucosamine pyrophosphorylase (UAP), and chitin synthase (CHS) are the major sites for chitin synthesis^3^. CHS activity influences chitin content^4^; trehalose, glycogen and glucose are used for the metabolic production of adenosine triphosphates and structural materials for chitin biosynthesis^3,5,6^; CHS activity is dependent on the presence of a divalent caution (Mg^2+^ or Mn^2+^)^7,8^.

**Figure 1:**
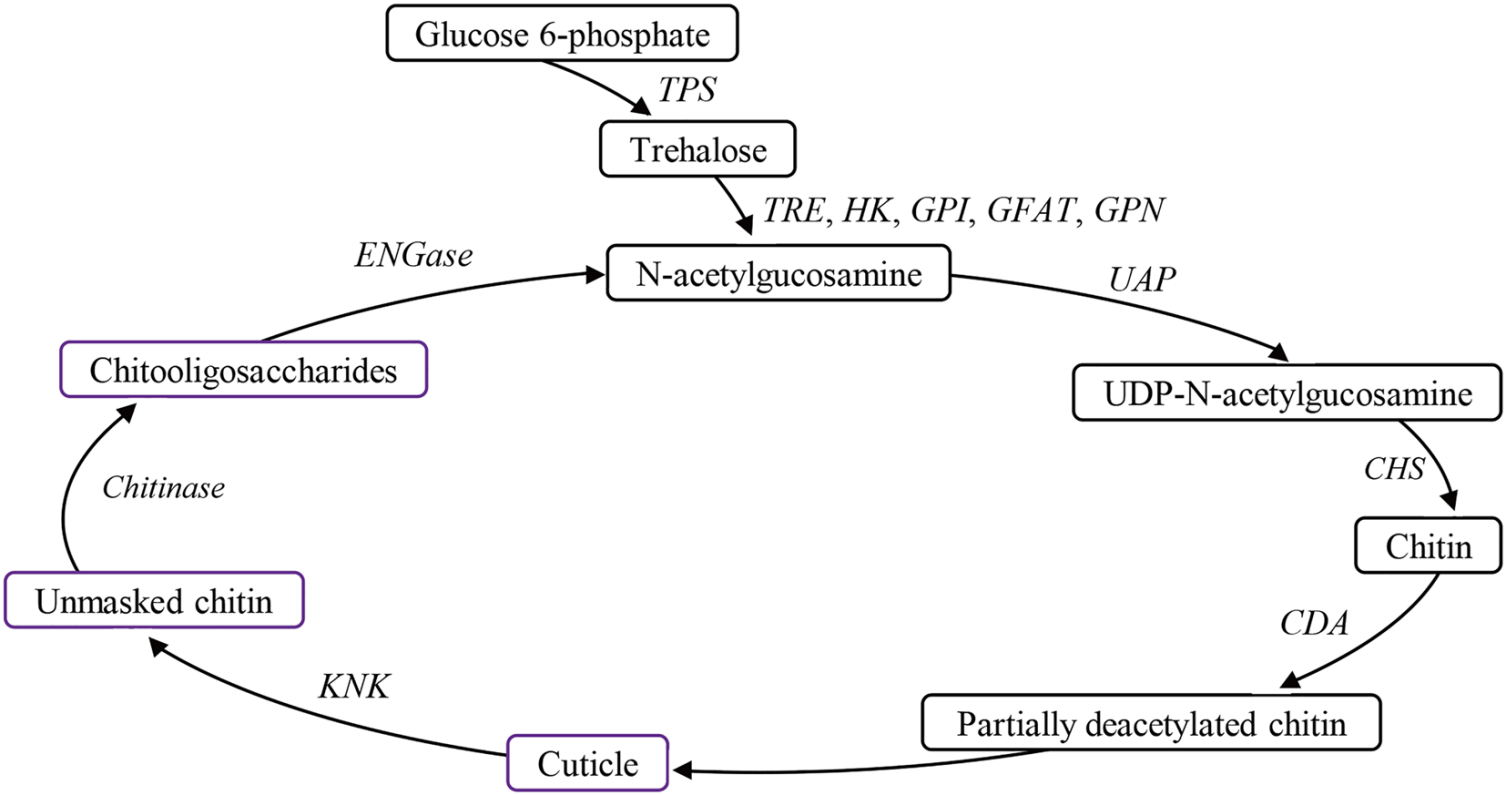
Key enzymes and genes involved in chitin biosynthesis and degradation in *A. pisum*. *TPS*, trehalose-6-phosphate synthase gene; *TRE*, trehalase gene; *HK*, hexokinase gene; *GPI*, glucose-6-phosphate isomerase gene; *GFAT*, glutamine-fructose-6-phosphate aminotansferasr gene; *GPN*, glucosamine-6-phosphate-N-acetytransferase gene, *UAP*; UDP-N-acetyglucosamine pyrophosphorylase gene; *CHS*, chitin synthase gene, *Cht1*, *2*, *3*, *5*, *6*, *7*, *8*, *10*, eight chitinase genes; *IDGF*, imaginal disc growth factor gene; *ENGase*, endo-β-N-acetylgucosaminidase gene.

Trehalose is a non-reducing disaccharide in insect hemolymph, which has many functions, such as facilitating carbohydrates absorption and acting as an energy source. It is the major substrate of chitin biosynthesis and also involves in a partial feedback mechanism to regulate feeding behavior and nutrient intake^5,7,9,10^. The biosynthesis of chitin involves eight enzymes or genes as shown in Fig. 1, such as trehalase (TRE; EC 3.2.1.28), hexokinase (HK), glucose-6-phosphate isomerase (GPI), GFAT, glucosamine-6-phosphate-N-acetyltransferase (GPN), phosphoacetyl glucosamine mutase (PGM), UAP, CHS^1,7,11^. Trehalose is formed by two glucose molecules linked by an α–α bond and widely exists in bacteria, fungi and plants^5,9^. It is synthesized by trehalose-6-phosphate synthase (TPS; EC 2.4.1.15) and trehalose-6-phosphate phosphatase (TPP; EC 3.1.3.12) in the fat body of insects, as well as in the integument, trachea, midgut, Malpighian tubule and muscle^12–15^. It is hydrolyzed by TRE to yield two glucose molecules. TPS, TPP and TRE are important in various physiological processes, such as flight^16^, resistance to environmental stress^17,18^, feeding behavior^10,19^ and chitin synthesis during molting^20–22^.

TPS is an important enzyme in trehalose synthesis, and regulates the insect physiology and behavior, including survival, molting, pupal metamorphosis and chitin metabolism^9,12,14,15,23^. Three categories of TPS genes (*TPS1*, *TPS2* and *TPS3*) have been found in insects^24^. RNAi of *TPS* decreased trehalose content and subsequent survival rate, produced three abnormal phenotypes (molting deformities, wing deformities, and molting and wing deformities) and changed the gene expression related to the chitin metabolism in *Nilaparvata lugens*^12,23^, *Leptinotarsa decemlineata*^14^, *Tribolium castaneum*^25^ and *Bactrocera minax*^26^. TRE regulates chitin metabolism in insects^27,28^ and is the first enzyme of trehalose catabolism and chitin biosynthesis^7,9,29,30^. Previous studies have shown that TREs have two forms, soluble trehalase (TRE1) and membrane-bound trehalase (TRE2), and silencing and/or mutation of *TREs* resulted in high mortality, abnormal phenotype, low chitin production, decreased food intake and changed expression of genes related to chitin metabolism^10,14,31–33^. Trehalose accumulation in *TRE* mutants increased larval mortality and affected intestinal integrity due to reduced chitin synthesis and low feeding rate of *Drosophila melanogaster*^10^. RNAi of *TRE* increased the mortality rate and the number of abnormal phenotypes, and decreased the chitin content of *Spodoptera exigua*, *T. castaneum* and *L. decemlineata*^14,31,33^. In *S. exigua*, RNAi of *SeTre-1* and *SeTre-2* produced three different lethal phenotypes (severe-abnormal, abdomen-abnormal and misshapen-wings), increased mortality rates and reduced the chitin content in the cuticle and midgut. Chen et al. found that the *SeTre-1* was highly expressed in the cuticle and Malpighian tubules, while *SeTre-2* was predominantly expressed in the tracheae and fat body^31^. In *L. decemlineata*, RNAi of *LdTRE1a* increased the mortality of the larvae and pupae, reduced the food intake and slowed down the growth^14^. Similarly, knockdown of *CHS* in *T. castaneum* decreased the survival and fecundity of the population resulting in lethal phenotype and low chitin content^4^. In *S. exigua*, the epithelial walls of the larval trachea expanded uniformly after the knockdown of *SeCHS-A*^34^. In *Toxoptera citricida*, nymphs failed to complete molting and entered the next developmental stage after the suppression of *TCiCHS* by feeding with plant-mediated dsRNA^35^. These results confirm that RNAi of *TPS* and *TRE* is a potential strategy for improving pest management practices^36^.

The pea aphid, *Acyrthosiphon pisum* (Hemiptera: Aphididae), is characterized by the complex life cycle, and displays two body color morphs (red and green) in field populations and each color morph is stable within each parthenogenetic clone^37,38^. Generally, *A. pisum* is considered to be one of the main agricultural pests. Damages by the aphids are caused not only by directly feeding on plant phloem sap but also by transmitting plant viruses^39^. Insecticide treatments remain a convenient way for *A. pisum* control, but excessively use insecticides has resulted in resistance and resurgence. Therefore, it is necessary to study more effective pest control methods.

In this study, in order to understand the functions of TPS and TRE genes in the chitin metabolic pathway of *A. pisum*, RNAi technology was used to silence the expression of *ApTPS* and *ApTRE*, and RT-qPCR was used to determine the expression level of genes related to chitin biosynthesis and degradation. As shown in Fig. 1, we selected ten chitin biosynthesis genes (*ApTRE*, *ApHK*, *ApGPI*, *ApGFAT*, *ApGPN*, *ApUAP*, *Apchs-2*, *ApGP* and *ApTPS*)^6^, two cuticle synthesis genes (chitin deacetylase gene (*ApCDA*) and Knickkopf protein gene (*ApKNK*))^40,41^ and ten chitin degradation genes (chitinase-like genes1, 2, 3, 5, 6, 7, 8, 10 genes, imaginal disk growth factor gene (*ApIDGF*) and endo-beta-N-acetylglucosaminidase gene (*ApENGase*))^42^. The phenotypes, chitin content and related mRNA expression levels of these genes were described, and the functions of *ApTPS* and *ApTRE* in the chitin metabolic pathway in the red and green morphs of *A. pisum* were clarified.

## Results

### RNAi of *ApTPS *and *ApTRE* change the activity of trehalase

**Figure 2:**
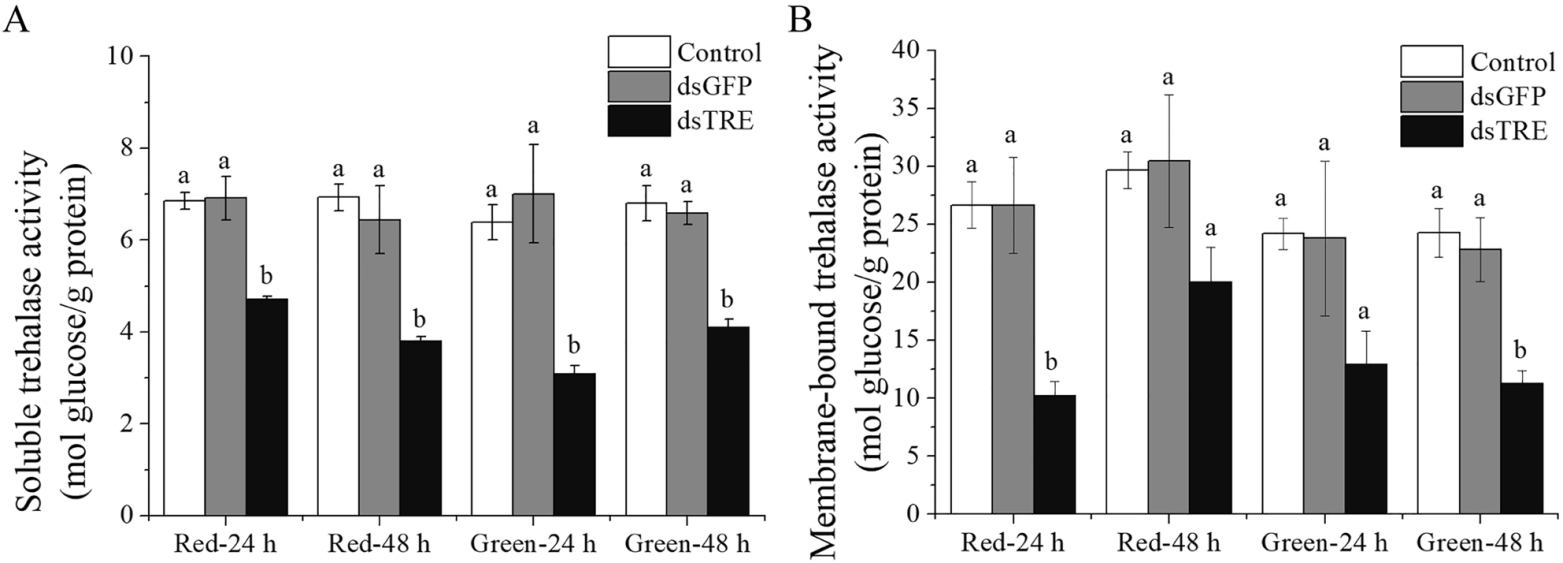
The enzyme activity of trehalase at 24 h and 48 h after RNAi. (**A**) The soluble trehalase activity after RNAi, (**B**) the membrane-bound trehalase activity after RNAi. *Control* the normal diet, *dsGFP* the GFP-dsRNA treatment, *dsTPS* the TPS-dsRNA treatment, *dsTRE* the TRE-dsRNA treatment, *Red* red morphs of *A. pisum*, *Green* green morphs of *A. pisum*. Each bar represents the Means ± SEM from three biological replicates with ten individuals mixed in each replicate. The data were analyzed using one-way analysis of variance (ANOVA), followed by the Tukey–Kramer test. The enzyme activity in the artificial diet group was designated as control, and different letters above the error bars indicate significant differences (*P* < 0.05).

We measured the activities of soluble trehalase (ApTRE1) and membrane-bound trehalase (ApTRE2) of *A. pisum* in each dsRNA-injected treatment group and found that the activities of ApTRE1 and ApTRE2 reduced in the dsTRE group compared with control and dsGFP groups (Fig. 2A,B).

### Effects of RNAi on phenotypes and chitin content

**Figure 3:**
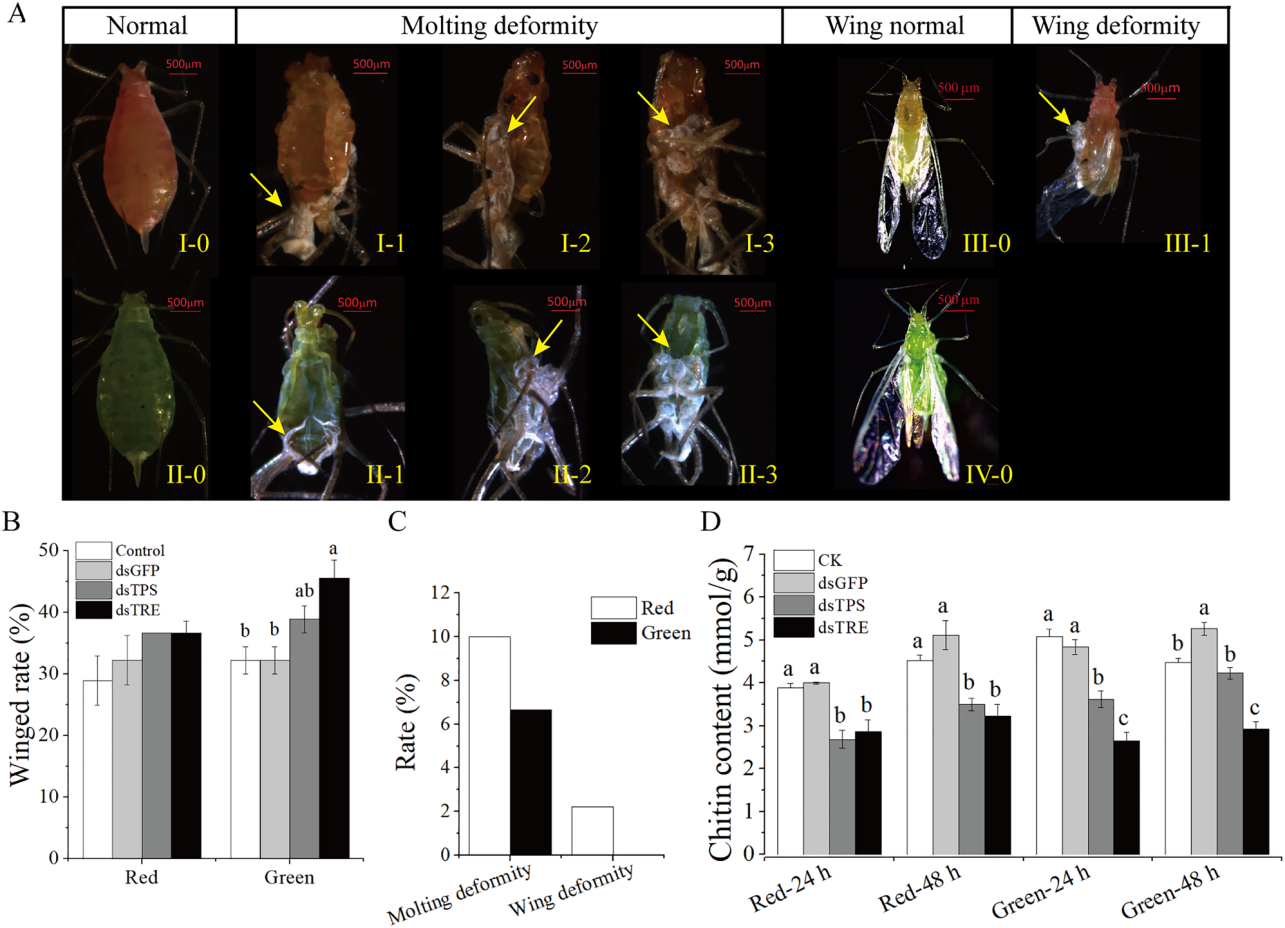
Effect of RNAi on phenotype and chitin content. (**A**) Representative phenotypes of *A. pisum* after RNAi. I-0 and II-0: normal phenotypes as control phenotype; II-3: abnormal phenotypes in the red morphs, the “molting deformity” phenotype; III-3: abnormal phenotypes in the green morphs, the “molting deformity” phenotype; III-0 and IV-0: normal winged and III-1: abnormal phenotype in the red morphs, the “wing deformity” phenotype. All abnormal insects in the nymph-adult stage were present. (**B**) The winged rate after RNAi. (**C**) The abnormal phenotype rates. (**D**) The chitin content after RNAi. Red, red morphs of *A. pisum*. *Green* green morphs of *A. pisum*, *CK* the normal diet, *dsGFP* the GFP-dsRNA treatment, *dsTPS* the TPS-dsRNA treatment, *dsTRE* the TRE-dsRNA treatment. Each bar represents the Means ± SEM from three biological replicates with ten individuals mixed in each replicate. The data were analyzed using one-way analysis of variance (ANOVA), followed by the Tukey–Kramer test. The different letters above the error bars indicate significant differences (*P* < 0.05).

As shown in Fig. 3A,B, compared with the control and dsGFP groups, the winged aphid rate was significantly higher in dsTRE of red morphs and dsTPS groups than in the dsTRE group of green morphs (Fig. 3B). The RNAi of *ApTRE* resulted in two abnormal phenotypes (molting deformity and wing deformity) in the red morphs (Fig. 3A,C), but only one abnormal phenotype (molting deformity) was observed in the green morphs when the molting deformities (the regions indicated by yellow arrows in Fig. 3A, I-1-3 and II-1-3) were compared with healthy aphids (Fig. 3A, I-0 and II-0). Similar effects were observed on wing deformities (the regions indicated by yellow arrow in Fig. 3A, III-1) compared with the normal wing phenotypes (Fig. 3A, III-0 and IV-0). However, no abnormal phenotypes were observed in the control and dsGFP and dsTPS groups. These results are consistent with previous studies that *TRE* silencing leads to abnormal phenotypes^14,31,33^, which may relate to the reduction of chitin synthesis.

We therefore analyzed the chitin content of whole aphids. These results indicated that chitin content decreased in *ApTPS* and *ApTRE* RNAi groups compared with control and dsGFP groups in two color morphs (Fig. 3D), but the chitin content significantly increased in the green morphs after dsGFP ingestion at 48 h. Moreover, knockdown of *ApTPS* reduced the relative chitin content by approximately 4–31% while knockdown *ApTRE* reduced more by 26–48% relative to those of the control group.

### Effect of RNAi of *ApTPS *and *ApTRE* on the expression of the genes related to chitin biosynthesis pathway

**Figure 4:**
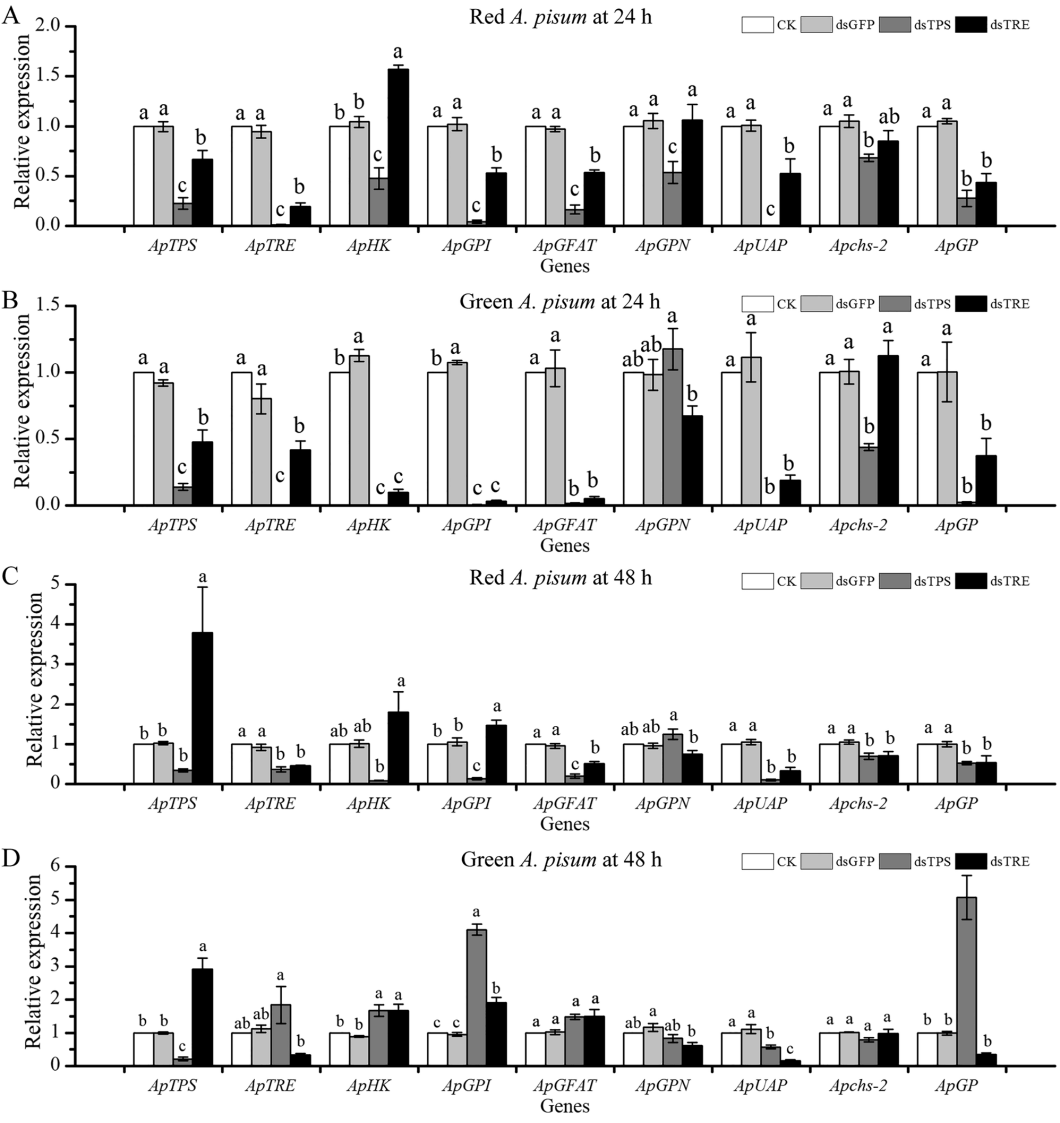
Effect of *ApTPS* and *ApTRE* RNAi treatments on the expression of the gens involved in the chitin biosynthesis. (**A**) in red morphs at 24 h, (**B**) in green morphs at 24 h, (**C**) in red morphs at 48 h, (**D**) in green morphs at 48 h. CK, the normal diet; dsGFP, the GFP-dsRNA treatment; dsTPS, the TPS-dsRNA treatment; dsTRE, the TRE-dsRNA treatment; *ApTPS*, trehalose-6-phosphate synthase gene; *ApTRE*, trehalase gene; *ApHK*, Hexokinase gene; *ApGPI*, glucose-6-phosphate isomerase gene; *ApGFAT*, glutamine-fructose-6-phosphate aminotansferasr gene; *ApGPN*, glucosamine-6-phosphate-N-acetytransferase gene; *ApUAP*, UDP-N-acetyglucosamine pyrophosphorylase gene; *Apchs-2*, chitin synthase gene 2; *ApGP*, glycogen phosphorylase gene. Each bar represents the Means ± SEM from three biological replicates and three technical replicates. The data were analyzed using one-way analysis of variance (ANOVA), followed by the Tukey–Kramer test. The mRNA expression level in the normal artificial diet group was designated as the reference control for the comparisons. The different letters above the error bars indicate significant differences (*P* < 0.05).

Our results showed that the expression levels of *ApTPS* and *ApTRE* were significantly lower in the dsTPS and dsTRE groups than in the control and dsGFP groups (Fig. 4). In addition, RNAi of *ApTPS* decreased *ApTPS* expression by approximately 70–80% compared with control groups, and RNAi of *ApTRE* decreased *ApTRE* expression by approximately 50–80%. These data suggested that the *ApTPS* and *ApTRE* genes were successfully targeted by their dsRNAs and the RNAi technique worked successfully.

The mRNA expression levels of the chitin biosynthesis pathway-related genes were detected after RNAi of *ApTPS* and *ApTRE*. As shown in Fig. 4, the expression of chitin biosynthesis-related genes was significantly altered by RNAi of *ApTPS*. In the red morphs, the expression of *ApTRE*, *ApHK*, *ApGPI*, *ApGFAT*, *ApUAP*, *Apchs-2* and *ApGP* was significantly down-regulated at 24 h and 48 h (Fig. 4A,C). The expression of *ApGPN* was significantly decreased at 24 h and then significantly increased at 48 h. Similarly, in the green morphs, the expression of *ApTRE*, *ApHK*, *ApGPI*, *ApGFAT*, *ApUAP*, *Apchs-2* and *ApGP* was significantly down-regulated at 24 h (Fig. 4B,D). The expression of *ApHK*, *ApGPI* and *ApGP* was significantly increased at 48 h, only *ApUAP* expression was significantly down-regulated at 48 h by RNAi of *ApTPS* (Fig. 4B,D).

RNAi of *ApTRE* significantly decreased the expression of *ApTPS*, *ApGPI*, *ApGFAT*, *ApUAP* and *ApGP* in the red morphs at 24 h (Fig. 4A). There was no difference (*P* > 0.05) in the expression of *ApGPN* and *Apchs-2* relative to those of the control group. The expression of *ApGFAT*, *ApUAP*, *Apchs-2* and *ApGP* was decreased significantly; the expression of *ApTPS* and *ApGPI* was increased significantly in the red morphs at 48 h (Fig. 4C), but there was no significant difference (*P* > 0.05) in the expression of *ApHK* and *ApGPN* relative to those of the control group. The expression of *ApTPS*, *ApHK*, *ApGPI*, *ApGFAT*, *ApUAP* and *ApGP* was decreased significantly in the green morphs at 24 h (Fig. 4B), but there was no significant difference (*P* > 0.05) in the expression of *ApGPN* and *Apchs-2*. However, the expression of *ApGPN*, *ApUAP*, *Apchs-2* and *ApGP* was decreased, and the expression of *ApTPS*, *ApHK*, *ApGPI* and *ApGFAT* was increased at 48 h (Fig. 4D).

### Effect of RNAi of *ApTPS *and *ApTRE* on chitinase and chitinase-like genes expression

**Figure 5:**
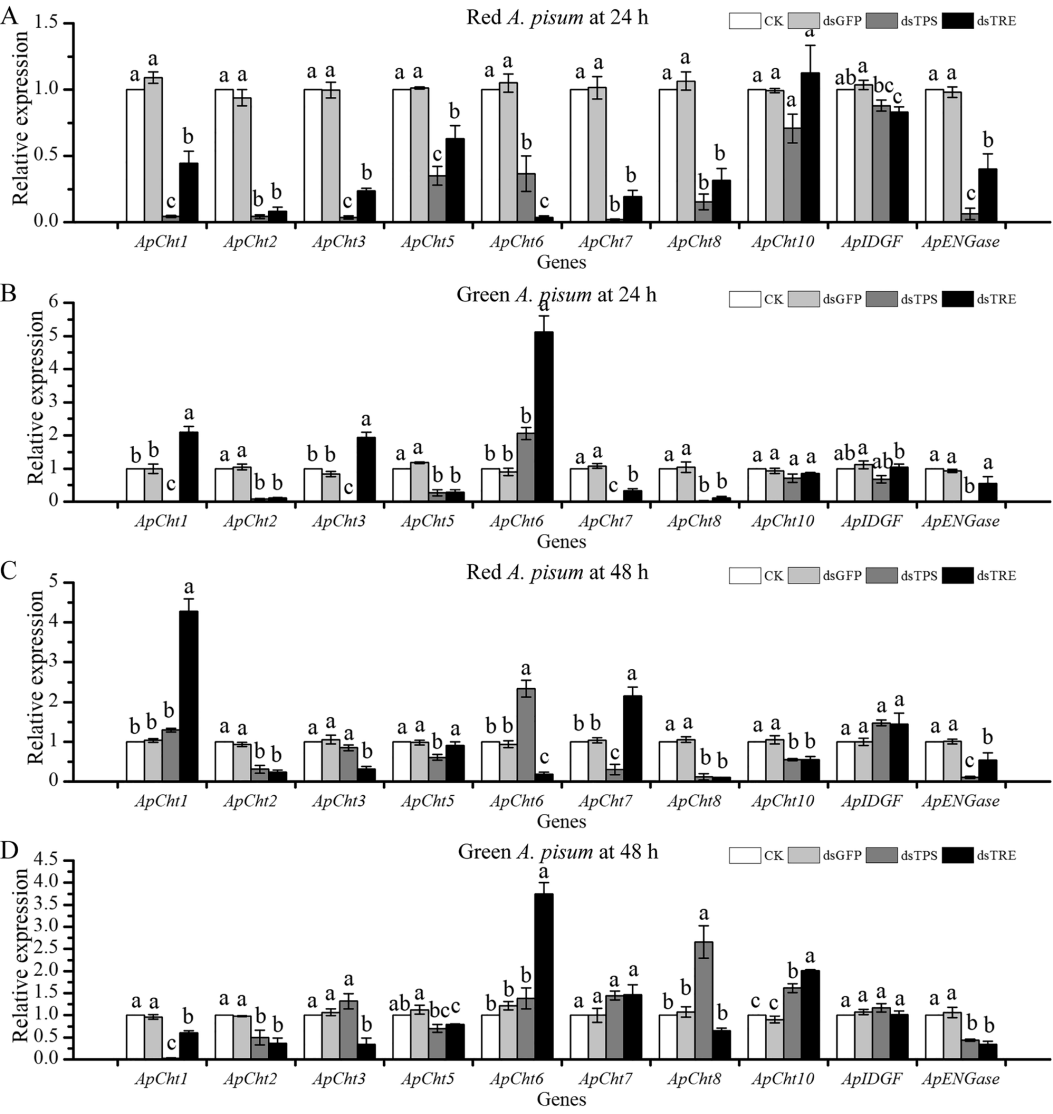
Effects of RNAi of *ApTPS* and *ApTRE* on relative expression of the chitinase and chitinase-like genes. (**A**) In red morphs at 24 h, (**B**) in green morphs at 24 h, (**C**) in red morphs at 48 h, (**D**) in green morphs at 48 h. CK, the normal diet; dsGFP, the GFP-dsRNA treatment; dsTPS, the TPS-dsRNA treatment; dsTRE, the TRE-dsRNA treatment; *ApCht1*, *2*, *3*, *5*, *6*, *7*, *8*, *10*; *ApIDGF*, Imaginal disc growth factor gene; *ApENGase*, Endo-β-N-acetylgucosaminidase gene. Each bar represents the Means ± SEM from three biological replicates and three technical replicates. The data were analyzed using one-way analysis of variance (ANOVA), followed by the Tukey–Kramer test. The mRNA expression level in the normal artificial diet group was designated as the reference control for the comparisons. The different letters above the error bars indicate significant differences (*P* < 0.05).

The mRNA expression levels of chitinase and chitinase-like genes were detected after RNAi of *ApTPS* and *ApTRE* (Fig. 5). RNAi of *ApTPS* decreased the expression of chitinase and chitinase-like genes in the red morphs at 24 h (Fig. 5A). In the red morphs, the expression of *ApCht1*, *2*, *3*, *5*, *7*, *8*, *10* and *ApENGase* was significantly decreased at 48 h (Fig. 5C), but the expression of *ApCht1*, *6* and *ApIDGF* was increased. In the green morphs, the expression of *ApCht1*, *2*, *3*, *5*, 7, *8*, *10*, *ApIDGF* and *ApENGase* was decreased at 24 h (Fig. 5B). However, the expression of *ApCht1*, *2*, *5* and *ApENGase* was significantly decreased at 48 h (Fig. 5D), but the expression of *ApCht6*, *7*, *8*, *10* and *ApIDGF* was increased.

RNAi of *ApTRE* significantly down-regulated the expression of *ApCht2*, *3*, *5*, *6*, *8*, *10* and *ApENGase* in the red morphs at 24 h and 48 h (Fig. 5A,C). The expression of *ApCht1* and *ApCht7* was decreased significantly at 24 h, and then significantly increased at 48 h (Fig. 5A,C). The expression of the *ApCht2*, *5*, *7*, *8* and *ApENGase* was decreased significantly in the green morphs at 24 h (Fig. 5B), but the expression of the *ApCht1*, *3* and *6* was significantly increased. The expression of *ApCht1*, *2*, *3*, *5*, *8* and *ApENGase* was decreased significantly at 48 h (Fig. 5B), but the expression of *ApCht6* and *ApCht10* was increased (Fig. 5D). There was no difference (*P* > 0.05) in the expression of *ApCht6* and *ApIDGF* between 24 and 48 h (Fig. 5B,D).

## Discussion

In our study, two abnormal phenotypes (molting deformities and wing deformities) were found in the aphid morphs after RNAi of *ApTRE* (Fig. 3A). However, no abnormal phenotypes were observed in the dsTPS, dsGFP and control groups. These abnormal phenotypes might be due to the remarkable decrease of chitin content (Fig. 3D), and in the dsTPS group the reduction of chitin synthesis did not reach the threshold for abnormal deformity. The abnormal phenotype rates are only 10% (Fig. 3C) considering that most of the genes were significantly changed by the RNAi of *ApTRE*. There seem some differences in the abnormal phenotype rates among insect species. It is reported that the silencing of *TPS* resulted in abnormal phenotypes in *L. decemlineata*^14^, *B. minax*^26^ and *N. lugens*^12^. Interestingly, the rates of molting and wing deformities were all less than 10% by RNAi of *TRE*, but the abnormal phenotypes rates were higher in *T. castaneum*^33^, *S. exigua*^31^ and *L. decemlineata*^14^.

The manipulation of chitin affected the growth and development of insects in previous studies^31,43–46^. Such as, suppression of *CHS* decreased the survival, fecundity, egg hatching ability and the number of insects molting^46^. Besides *ApTPS* and *ApTRE* suppression decreased and increased the content of trehalose (data not shown), respectively. It was reported that trehalose content affected the survival and feed behavior of insects^14,15,24,31^. Trehalose is known to be a precursor of chitin synthesis and would affect the chitin biosynthesis pathway. Our results showed that RNAi of *ApTPS* and *ApTRE* down-regulated the expression levels of chitin biosynthesis-related genes (Fig. 4A,B) as reported in *N. lugens*^12^ and *T. castaneum*^25,33^. In contrast, in the *N. lugens*, the expression of *NlGFAT*, *NlGNPNA* and *NlUAP* was significantly increased by knockdown of two *NlTPS*^12^. These differences may be due to differences between the species genome. We also found that the expression of glycogen phosphorylase (*ApGP*) significantly decreased after RNAi of *ApTPS* and *ApTRE* (Fig. 4) as in *N. lugens*^13^, supporting previous studies that *ApTPS* and *ApTRE* also regulate chitin synthesis by influence the expression of glycogen phosphorylase (GP) and glycogen synthase (GS) genes because insects must accumulate glycogen before entering diapause^33,47,48^. Thus, RNAi of *ApTPS* and *ApTRE* may enhance glycolysis by activating glycogen phosphorylase and then influence glycogen metabolism. Our results showed that the silencing of *ApTPS* and *ApTRE* altered the expression levels of chitin degradation-related genes (Fig. 5), similar results were shown in *N. lugens*^12,49^, and thus influence aphid development as other studies showed that chitin-degrading enzymes play an important role in growth and development, especially during larval molt and pupation^1,15^.

**Figure 6:**
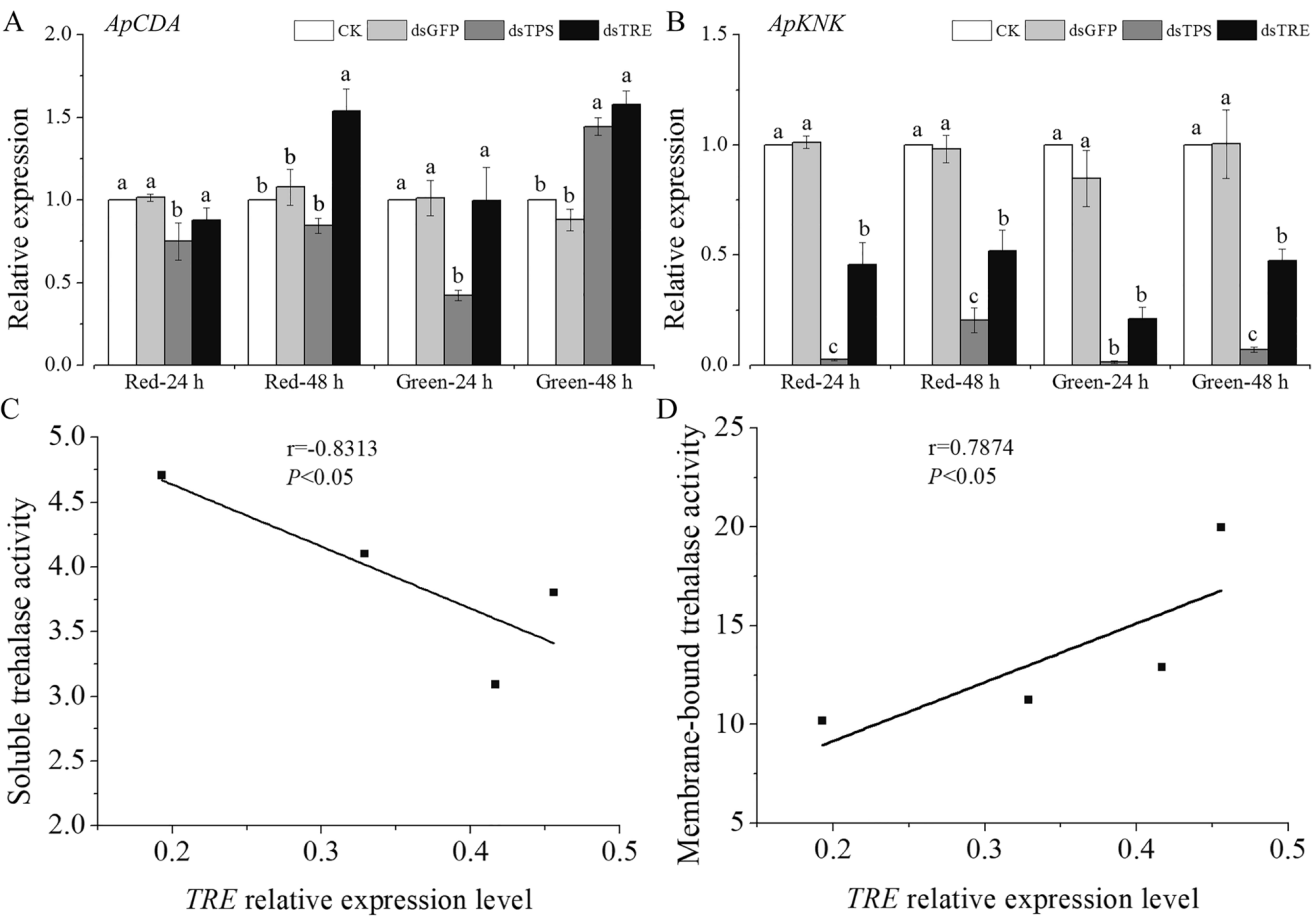
The expression levels of *ApCDA* and *ApKNK* at 24 h and 48 h after RNAi of *ApTPS* and *ApTRE*. (**A**) Chitin deacetylase gene, (**B**) Chitin-binding protein Knickkopf gene, (**C**, **D**) The correlation analyses between the trehalase activity and the expression of *ApTRE*. Each point in the figure represents a sample, and the *P*-values and the correlation coefficient r are presented. Bivariate correlations between variables were calculated using Spearman’s rank correlation coefficients. *CK*, the normal diet; *dsGFP*, the GFP-dsRNA treatment; *dsTPS*, the TPS-dsRNA treatment; *dsTRE*, the TRE-dsRNA treatment. Each bar represents the Means ± SEM from three biological replicates and three technical replicates and the data were analyzed using one-way analysis of variance (ANOVA), followed by the Tukey–Kramer test. The mRNA expression level in the artificial diet group was designated as control, different letters above the error bars indicate significant differences (*P* < 0.05).

Chitin deacetylase (CDA) is found in the cuticles and peritrophic matrix of insects. It is conceivable that partial deacetylation may render matrix chitin more resistant to hydrolysis by endochitinases^6^. Our results showed that RNAi of *ApTPS* inhibited *ApCDA* expression in the red and green morphs at 24 h, but promoted *ApCDA* expression in the green morphs at 48 h (Fig. 6A). The expression of *ApCDA* was also promoted by RNAi of *ApTRE* at 48 h (Fig. 6A). In addition, the Knickkopf protein (KNK) is a cuticular protein that protects chitin from chitinases and organizes it into laminae^40^. In our study, we showed that the *ApKNK* expression significantly decreased after RNAi of *ApTPS* and *ApTRE* (Fig. 6B). Previous studies have demonstrated that knockout of *SpCDA1* and *TcKNK* increased molting difficulty and cause high larval mortality in *Stegobium paniceum* and *T. castaneum*^6,40,41,50^. Interestingly, the expression of *ApCDA1* and *ApKNK* at 48 h was significantly higher compared with those at 24 h (Fig. 6A,B), which may be a feedback regulation between lower chitin content and the up-regulated expression of chitin metabolism-related genes (Figs. 4D and 5D). The enzyme activities of ApCDA and ApKNK were increased for protective cuticle degradation. We also found that the expression of some chitinase and chitinase-like genes were restored and even enhanced at 48 h. Interestingly, Yang et al. also found similarity trends at 72 h after RNAi of *TPS*^21^. In addition, the increase in the expression, restoration and enhancement of the chitin synthesis-related genes *ApTPS*, *ApHK* and *ApGPI* by RNAi of *ApTRE* (Fig. 4C,D) may be a possible feedback mechanism to improve stress resistance, protect cellular structures and present extensive gene duplication^51–53^, accelerating the degradation of old cuticle to provide precursor for the synthesis of new cuticle as Fig. 1 shows. Figure 6C, D showed there was a negative correlation of the *ApTRE* expression level with the activity of ApTRE1 (Fig. 6C; r = − 0.8313, *P* < 0.05), and a positive correlation with the activity of ApTRE2 (Fig. 6D; r = 0.7874, *P* < 0.05).

We found five *ApTRE* gene isomers (XM_001950229.4, XM_003245847.4, XM_016808594.2, XM_003247977.3 and XM_029491831.1) and two *ApTPS* gene isomers (XM_003244776.4 and XM_001943581.5) from the aphid genome (Supplementary Fig. 1). RNAi experiments of *ApTRE* reduced trehalase activities (Fig. 2), suggesting there is no compensation between isomers in *A. pisum* as reported in *Nilaparvata*^27^. Our *ApTRE* primer sequences match 100% to two of five *ApTRE* isomers (XM_001950229.4 and XM_003245847.4) and 70% to other three *ApTRE* isomers (XM_016808594.2; XM_003247977.3 and XM_029491831.1). The comparison between the sequence of the PCR product using our primer sequences with these gene sequences (Supplementary Fig. 2) suggested that under our PCR condition all gene copies could be amplified. The RT-qPCR results and the activity of trehalases after RNAi reflect a total gene expression and enzymatic activities (Fig. 2). Bansal et al. by phylogenetic analysis suggestion the *ApTRE* gene (XM_001950229.4) was soluble trehalase^54^. It is not clear whether XM_003245847.4 could be a membrane-bound form.

We found some different effects of RNAi of *ApTPS* and *ApTRE* on the expression of chitin metabolism-related genes between red and green morphs (Figs. 4 and 5). Two diagrammatic representations, related to insect chitin biosynthesis and degradation pathways, are drafted in Supplementary Fig. 5 for the 48 h expression data, and Supplementary Table 3 for the 24 h data. The figure also summarizes the differences between red and green morphs. Such as, RNAi of *ApTRE* increased *ApHK* expression in the red morphs at 24 h, but decreased by 90% in the green morphs (Fig. 4A,B). This difference might show color polymorphism of *A. pisum*, and may be related to local adaptation and ecological speciation^55^. In addition, in the green morphs, the content of chitin at 48 h (Fig. 3D) and the expression of *ApHK* and *ApGPI* at 24 h (Fig. 4B) were significantly higher in the dsGFP group than in the control group. This may be due to the metabolic disorder induced by the deleterious substances in artificial diet. It has been found that the artificial diet influenced the growth, development and reproduction^56^.

RNAi technology is a promising mean to study gene function and could be used as a novel control strategy for agricultural crop pests. Silencing of *ApTPS* and *ApTRE* affects the growth and development of the aphids and proves that trehalose plays a vital role in chitin metabolism and aphid phenotypes. These results can deepen our understanding of the biological functions of *ApTPS* and *ApTRE* in *A. pisum*, lay a foundation for a better understanding into the molecular mechanisms of insect metamorphosis, and potentially using them as candidate genes in agricultural pest management.

## Materials and methods

### Insect and culture conditions

Clones of red and green morphs of *A. pisum* were established from single virginiparous females. They were collected in 2017 from the same plant of *M. sativa* in Lanzhou, China, and were reared on the fava bean *Vicia faba*. All plants and aphid cultures were reared at 20 ± 1 °C, 70 ± 10% relative humidity, with a photoperiod of 16 h L:8 h D. Mature aphids were put on a fava bean leaf for 12 h and the resulting neonate nymphs, 0–12 h old, were used throughout the experiment.

### RNA isolation and first-strand cDNA synthesis

Total RNA was isolated using TRizol reagent (BBI Life Sciences, Shanghai, China) following manufacturer’s instructions. The total quantities of extracted RNA were assessed using a micro-volume UV spectrophotometer (Quawell Q5000, Quawell, USA). The RNA integrity was further confirmed by 1% formaldehyde agarose gel electrophoresis. Total RNA was dissolved in 50 μL DEPC-water and stored at − 80 °C. The first-strand cDNA was synthesized using a First-Strand cDNA Synthesis kit (BioTeke, Beijing, China), stored at − 20 °C and used for subsequent experiments.

### Cloning of TPS and TRE cDNAs

The primer sets, TPS-F/R and TRE-F/R, used for cloning of *ApTPS* and *ApTRE* were designed using the primer software Primer 5.0 (Premier Biosoft, Palo Alto, CA, USA) based on the TPS gene sequence (GENBANK accession: XM_001943581.5), and the TRE gene sequence (GENBANK accession: XM_003245847.4) of the *A. pisum*. The primers for the Green Fluorescent Protein (pET28a-EGFP, miaolingbio, Wuhan, China) was referenced in Yang^21^. All sequences of these primers used are listed in Supplementary Table 1. The component of the PCR mixture included 1.0 μL of the template (1 ng/μL), 12.5 μL 2 × Power Tap PCR MasterMix (BioTeke, Beijing, China), 1.0 μL of each primer (10 μmol/μL), and 9.5 μL Rnase-free H_2_O for a final volume of 20 μL. The PCR conditions were pre-denaturation at 95 °C for 5 min, 35 cycles at 95 °C for 45 s, at 55 °C for 45 s and at 72 °C for 1 min, then 10 min at 72 °C for a final extension. The PCR products were subjected to 1.0% agarose gel electrophoresis and purified by the DNA gel extraction kit (BioTeke, Beijing, China). The purified PCR product was ligated into the pMD18-T vector (TaKaRa, Dalian, China) and sequenced by Tsing Ke Biological Technology (Tsing Ke Biological Technology, Beijing, China) using the dideoxynucleotide method. The lengths of the resulting *ApTPS*, *ApTRE,* and *GFP* genes were 421 bp, 416 bp, and 688 bp, respectively. Next, we aligned five *ApTRE* isomers and two *ApTPS* isomers with our primers and the sequences of the PCR products (Supplementary Fig. 1), respectively.

### dsRNA synthesis and feeding

Three pairs of primers (dsTPS-F/R, dsTRE-F/R and dsGFP-F/R), with the T7 RNA promoter sequence flanking at the 5′-end of each gene, were designed and synthesized (Supplementary Table 1) and used to make the templates for in vitro dsRNA transcription via PCR. The dsRNAs were synthesized using the TranscriptAid T7 High Yield Transcription Kit (Thermo Scientific, Wilmington, DE, USA) according to manufacturer’s protocol^41^. The size of the dsRNA products was confirmed by electrophoresis on a 1.5% agarose gel and the content was assessed using a micro-volume UV spectrophotometer.

The artificial diet was made according to the following procedures of previous studies^57,58^ with sucrose (845 mM), l-amino acids, Vitamins and others. The l-amino acids (mM) include Ala (20.06), β-Ala (0.70), Asn (14.06), Asp (19.88), Cys (2.44), Glu (10.15), Gln (30.49), Gly (22.19), His (6.94), Leu (20.06), Lys (19.22), Met (4.85), Orn (0.56), Phe (10.29), Pro (11.23), Ser (11.83), Thr (10.67), Try (2.09), Tyr (2.13) and Val (16.29). The vitamins (mg/100 mL) include p-aminobenzoic acid (10), l-ascorbic (100), biotin (0.1), d-calcium pantothenate (5), choline chloride (50), folic acid (1), I-inositol (42), nicotinamide (10), pyridoxin HCl (2.5), riboflavin (0.5) and thiamine di-HCl (2.5). Other components (mg/100 mL) are CuSO_4_·5H_2_O (0.47), FeCl_3_·6H_2_O (4.45), MnCl_2_·4H_2_O (0.65), NaCl (2.54), ZnCl_2_ (0.83), calcium citrate (10), cholesteryl benzoate (2.5), MgSO_4_·7H_2_O (242) and KH_2_PO_4_ (250). The diet was filtered through a 2 μm membrane, dispensed in 1.0 mL aliquots, and stored at − 20 °C before the artificial diet feeding assay.

Glass vials (2.5 cm in diameter) were sterilized and used for the aphid artificial double-membrane feeding assay. Briefly, one opening of glass vials was completely sealed with parafilm. A given volume of test samples containing either nuclease-free water or dsRNA was added to the 1.0 mL artificial diet for a final concentration of 400 ng/μL. Seventy microliters of the artificial diet with either *dsTPS*, *dsTRE*, or *dsGFP* were sandwiched between two layers of the parafilm membrane^58^.

Fifteen 3-day-old aphids were introduced into one vial with a fine paintbrush. Then, another vial with the diet sandwich was closed with the aphid containing vial by a piece of sterilized gauze. The control group was only fed with the artificial diet without dsRNA. The artificial diet was replaced every other day to prevent dsRNA degradation. After 4 days, all surviving morphs from each treatment were selected and divided randomly into three replicates groups, and then transferred to fresh bean leaf discs. The collections were done at 24 h and 48 h after feeding on plant to alleviate the adverse effects of the artificial diet on treated aphids and allow the aphids to adapt feeding on the plants. Previous studies have shown that RNAi effects can be remained from the parents subjected to RNAi in their progenies^59^. Abdellater et al. found that RNAi had a prolonged impact and remained significantly effective in the six subsequent generations^60^. Mutti et al. also found that *Coo2* (Salivary gland transcript) expression dropped dramatically in 3 days after injection with dsRNA^61^.

### Phenotype observations

Phenotypes of 30 wingless aphids in each treatment group were observed and analyzed after the RNAi treatments every 12 h until they became adults and began to produce young nymphs. Three replicates were set for each treatment.

### Trehalase activity assay

Insects were subjected to soluble and membrane-bound trehalase activity analyses at 24 h and 48 h after the 4-day RNAi treatment. Trehalase activity was assayed according to the method described by Shen et al.^22^, with some modifications. Briefly, ten aphids were homogenized in phosphate-buffered saline (PBS: 130 mM NaCl; 7 mM Na_2_HPO_4_·2H_2_O; 3 mM NaH_2_PO_4_·2H_2_O; pH 7.0), then centrifuged at 1000 × *g* for 20 min at 4 ℃. Subsequently, 450 μL of the supernatant was centrifuged again at 10,000 × *g* for 20 min at 4 ℃. After superspeed centrifugation, the supernatant was directly used to determine the soluble TRE activity and protein content, while the sediment was re-suspended in PBS to evaluate the membrane-bound TRE activity and protein content. The measurement of trehalase activities was based on the rate of glucose released from trehalose. Either the supernatant (TRE1 activity) or the suspension (TRE2 activity) obtained from ultracentrifugation (70 μL) was uniformly mixed with 40 μL of 40 mM trehalose (Sangon Biotech, Shanghai, China) and 180 μL of PBS. The mixture was then incubated at 37 ℃ for 60 min and centrifuged at 12,000 × *g* for 10 min at 4℃. The resulting supernatant (20 μL) was used to determine the TRE1 and TRE2 activities using the glucose assay kit (Solarbio Biochemical Assay Division, Beijing, China) according to the manufacturer’s protocols. The protein concentration was determined using the BCA Protein Assay Kit (Sangon Biotech, Shanghai, China) according to the manufacturer's instruction. Three replicates were set for each RNAi treatment and the control.

### Measurements of chitin content in RNAi aphids

Ten individuals were homogenized with 1 mL phosphate-buffered saline (PBS: 130 mM NaCl, 7 mM Na_2_HPO_4_·2H_2_O, 3 mM NaH_2_PO_4_·2H_2_O; pH 7.0). The total chitin was extracted from the aphid body and analyzed according to the method described by Xia and Shen^62^ with sight modifications. Briefly, the homogenate was centrifuged at 1800 × *g* for 15 min at 25 ℃. The supernatant was discarded and the pellet was suspended in 400 μL of 3% sodium dodecyl sulfate (SDS), centrifuged at 1800 × *g* for 5 min at 100 ℃, and then centrifuged again at 1800 × *g* for 10 min at 25 ℃. To deacetylate chitin, the pellet was redissolved in 0.3 mL of KOH (14 mol/L) and incubated in dry baker at 130 ℃ for 1 h. Celite and different concentrations of absolute alcohol were added to the samples to obtain the insoluble chitosan. 100 μL of chitosan extract solution, 50 μL of 10% KHSO_4_ and 50 μL of 10% NaNO_2_ were mixed and incubated at 25 ℃ for 15 min to deaminate the glucosamine residues and depolymerize the chitosan. After the incubation with 40 μL of 12.5% NH_4_SO_3_NH_2_ at 25 ℃ for 15 min, 40 μL of 3-methyl-2-benzothiazolone hydrazone hydrochloride hydrate (MBTH, 5 g/L) was mixed and incubated at 25℃ for 5 min. Then, 40 μL of 0.83% FeCl_3_ was added and incubated at 25 ℃ for 10 min. Finally, the chitin content was measured in a 96-well micro plate in a total reaction volume of 160 μL for each sample per micro-plate well. Changes of absorbance were measured under 650 nm in a micro plate spectrophotometer (Bio Tek ELX800UV, Winsooski, VT, USA). A control reaction treated with double distilled water was included for comparison. The chitin content was calculated based on the established standard concentration curve of d-(+)-Glucosamine HCl (Solarbio, Beijing, China). Three replicates were set for each treatment.

### Quantification of mRNA expression levels

Aphids were immediately frozen in liquid nitrogen and total RNA was isolated from seven whole aphids. The first-strand cDNA was synthesized from total RNA using a First-Strand cDNA Synthesis kit (BioTeke, Beijing, China). The RT-qPCR analysis was carried out in 96-well 0.1-mL Block plates using a QuantStudio™ 5 system (Thermo Scientific, Wilmington, DE, USA). Each reaction contained 1.0 μL of the template (1 ng/μL), 10.0 μL of 2 × Plus SYBR real-time PCR mixture (BioTeke, Beijing, China), 0.5 μL of each primer (10 μmol/μL), 8 μL of EDPC-ddH_2_O, and 0.5 μL of 50 × ROX Reference Dye in a final volume of 20 μL. The RT-qPCR condition was pre-denaturation at 94 °C for 2 min, and then 40 cycles at 94 °C for 15 s and at 55–62 °C for 30 s. After each reaction, a melting curve analysis (denatured at 95 °C for 15 s, annealed at 60 °C for 1 min, and denatured at 95 °C for 15 s) was conducted to ensure consistency and specificity of the amplified product. Three biological replicates and three technical replicates were set in the RT-qPCR analyses for each treatment. Quantification of the transcript level was conducted according to the ${2}^{-\Delta \Delta Ct}$ method^63^. The RT-qPCR primers of chitin metabolism-related genes were designed to determine the expression of the corresponding homologous genes, including *ApTRE*, *ApHK*, *ApGPI*, *ApGFAT*, *ApGPN*, *ApUAP*, *Apchs-2*, *ApGP*, *ApTPS*, *ApCht1*, *2*, *3*, *5*, *6*, *7*, *8*, *10*, *ApIDGF*, *ApENGase*, *ApCDA* and *ApKNK*, and the ribosomal protein L27 gene (*rpL27*) was used as the reference gene^61^ (Supplementary Table 2).

### Statistical analysis

The enzyme activity, abnormal phenotype rates, mRNA expression level and chitin content of the aphids fed with in the normal artificial diet without dsRNAs were designated as control. All data were represented as Means ± SEM of three replicates per each treatment and analyzed using a one-way analysis of variance (ANOVA) followed by Tukey–Kramer test. Bivariate correlations between variables were calculated using Spearman’s rank correlation coefficients. *P* values of < 0.05 were considered statistically significant. All statistical analyses were performed using IBM SPSS 19.0, Origin 8.5. Excel 2010 were used to construct the histograms. The different letters indicate a significant difference in mRNA levels between the artificial diet group (CK) and the dsRNA-ingested group measured simultaneously. The down/up-regulation of the genes related to chitin metabolism at 24 h after the RNAi treatments were showed in Supplementary Table 3.

## Supplementary Information

Supplementary Information.
