# Supplementary material for: RNA interference of trehalose-6-phosphate synthase and trehalase genes regulates chitin metabolism in two color morphs of Acyrthosiphon pisum Harris
Source: Sci Rep. 2021 Jan 13;11:948. doi: 10.1038/s41598-020-80277-2 (PMC7806880; doi:10.1038/s41598-020-80277-2)
Supplement: Supplementary file 1 — Supplementary Information. [file 41598_2020_80277_MOESM1_ESM.pdf]

RNA interference of trehalose-6-phosphate synthase and trehalase genes regulates chitin metabolism in two color morphs of *Acyrthosiphon pisum* Harris

Guang Wang[1,2], Yuping Gou[1,2], Sufan Guo[1,2], Jing-Jiang Zhou[1,2], and Changzhong Liu[1,2,*]

[1] College of Plant Protection, Gansu Agricultural University, Lanzhou 730070, Gansu, China
[2] Biocontrol Engineering Laboratory of Crop Diseases and Pests of Gansu Province, Lanzhou 730070, Gansu, China

* corresponding author: liuchzh@gsau.edu.cn

genes	Primer set	Primers sequence (5'-3')	Product Size (bp)
ApTPS	TPS-F	GGATGCAACCTACCACCCAA	421
	TPS-R	CCCGGTAGTGATACGTCAGCT	
ApTRE	TRE-F	GGCGTGGTTTGACTGGGATA	416
	TRE-R	CAACCAAAACCTGTCTGCGG	
GFP	GFP-F	AAGGGCGAGGAGCTGTTCACCG	688
	GFP-R	CAGCAGGACCATGTGATCGCGC	
dsTPS	dsTPS-F	TAATACGACTCACTATAGGGGGATGCAACCTACCACCCAA	421
	dsTPS-R	TAATACGACTCACTATAGGGCCCGGTAGTGATACGTCAGC	
dsTRE	dsTRE-F	TAATACGACTCACTATAGGGGGCGTGGTTTGACTGGGATA	416
	dsTRE-R	TAATACGACTCACTATAGGGCAACCAAAACCTGTCTGCGG	
dsGFP	dsGFP-F	TAATACGACTCACTATAGGGAAGGGCGAGGAGCTGTTCACCG	688
	dsGFP-R	TAATACGACTCACTATAGGGCAGCAGGACCATGTGATCGCGC	

Supplementary Table 1. PCR primers used for dsRNA synthesis in this study. T7 RNA promoter sequence: TAATACGACTCACTATAGGG

Gene	GeneBank number	Primer set	Forward (5'-3')	Reverse (5'-3')	Product size (bp)
ApTPS	XM_001943581.4	QTPS	ACCGACCGTGCCAATTTCGTTT	CGAAACCAAGGGGGCTGTTTG	136
ApTRE	XM_003245847.4	QTRE	CGCATTGTACGCTGGTCATT	AGTTGGACCCTGAGACTCCT	80
ApHK	XM_003242192.3	QHK	CCTTGAAGGTTCTGAGCGAGATGG	GGATACGCACTCACAGATGTACCG	86
ApGPI	XM_022320851.1	QGPI	CTGCTAAAGATGAATCCAGTGTTG	ACCAAAGTGAGTAACGTCCAC	138
ApGFAT	XM_003246505.3	QGFAT	AATACATTCAGACCATCCCCG	TCCCTGGATTTCGGTGTTAAG	115
ApGPN	NM_001162048.2	QGPN	AGCGTCACTCAGCAGATGTATTCG	AATTCTACCGCGTTGTCCACAGAG	168
ApUAP	XM_015507631.1	QUAP	CTGCTGCAGGCTACACTCAA	AGCTTCTAGGCACCAACTGC	119
Apchs-2	XM_003247469.3	QCHS	TGGCTTCTATCTCAAACGTGG	GTCCCATCTCCTGTTCATTCC	144
ApGP	XM_022305439.1	QGP	GGTCTGGCAGCTTATGGTTACGG	CGTCTGGTTCTTCGGTCTGTTCAC	91
ApCHT1	HM001231.1	QCHT1	GTCAAGCACCACTGGTCCTAACG	GGCTGCTAACCAATCATCGACTCC	100
ApCHT2	XM_001943003.4	QCHT2	ACGTCATCACCGCACTACAACAC	GCTTCGTCGTCGTCGGCTTC	109
ApCHT3	XM_015513030.1	QCHT3	GGTGGAGTTATGATATGGTCTGTAG	GTAGCTTCATATGGTCCGTCG	143
ApCHT5	XM_016803409.1	QCHT5	CAGCGAGCACGACCAATGTAGC	CCGTTGTTGTCGTTGGCGTTAATG	180
ApCHT6	XM_008184636.2	QCHT6	AATCGTGGTCCGTGACCTAA	TGCGGTTCTGAATGTCCAAG	168
ApCHT7	XM_008185544.2	QCHT7	CCGTAGAAGGTGTGCCACAAGAC	GATCCAGTGTCCATTGTCCAGCAG	150
ApCHT8	XM_001945435.4	QCHT8	CCGGCATATCAGCGCAGATCAC	ACAATCTTGGTCCTTCAGCCATCG	183
ApCHT10	XM_022323068.1	QCHT10	GGTGGAGTTATGATATGGTCTGTAG	TTCCTGTGGCTTCATATGGTC	148
ApIDGF	NM_001168671.1	QIDGF	GGCACGTATACTAAGACCGAAG	GCAAACGGAAAGCATAGGTTC	148
ApENGase	XM_001949910.4	QENGase	ACGGTGAAGCAGTTATAGGC	GGGAGTCATAGGGCTTTAATGC	150
ApCDA	XM_003244847.3	QCDA	TCATTACACGAACCCACACG	TTGTTCGTCACCTTCGTCAG	148
ApKNK	XM_001950790.4	QKNK	TTATGGGGGCAACAACCCTC	AACATAATGGGCCCGCAGAA	179
rpL27	CN584874	QrpL27	CCGAAAAGCTGTCATAATGAAGACC	GGTGAAACCTTGTCTACTGTTACATCTTG	231

Supplementary Table 2. RT-qPCR primers used in this study. The abbreviations are *ApTPS*: trehalose-6-phosphate synthase gene, *ApTRE*: trehalase gene, ApHK: hexokinase gene, *ApGPI*: glucose-6-phosphate isomerase gene, *ApGFAT*: glutamine-fructose-6-phosphate aminotansferasr gene, *ApGPN*: glucosamine-6-phosphate-N-acetytransferase gene, *ApPGM*: phosphoacetyglucosamine mutase gene, *ApUAP*: UDP-N-acetyglucosamine pyrophosphorylase gene, *Apchs-2*: chitin synthase gene 2, *ApGP*: glycogen phosphorylase gene, *ApCht1*, *2*, *3*, *5*, *6*, *7*, *8* and *10*: eight chitinase-like genes 1, 2, 3, 5, 6, 7, 8 and 10 genes, *ApIDGF*: imaginal disk growth factor gene, *ApENGase*: endo-beta-N-acetylglucosaminidase gene, *ApCDA*: chitin deacetylase gene, *ApKNK*: knickkopf protein gene and *rpL27*: ribosomal protein L27 gene.

Pathway	Gene	Red *A. pisum*				Green *A. pisum*			
		dsTPS		dsTRE		dsTPS		dsTRE	
		24 h	48 h	24 h	48 h	24 h	48 h	24 h	48 h
Chitin biosynthesis	*ApTPS*	↓	↓	↓	×	↓	↓	↓	↑
	ApTRE	↓	↓	↓	↓	↓	×	↓	↓
	ApHK	↓	↓	↑	×	↓	↑	↓	↑
	ApGPI	↓	↓	↓	↑	↓	↑	↓	↑
	ApGFAT	↓	↓	↓	↓	↓	↑	↓	×
	ApGPN	↓	↑	×	×	×	×	×	↓
	ApUAP	↓	↓	↓	↓	↓	↓	↓	↓
	Apchs-2	↓	↓	×	×	↓	↓	×	×
	ApGP	↓	↓	↓	×	↓	↑	↓	↓
Cuticle synthesis	*ApCDA*	↓	↓	×	↑	↓	↑	×	↑
	ApKNK	↓	↓	↓	↓	↓	↓	↓	↓
Chitin degradation	*Cht1*	↓	↑	↓	↑	↓	↓	↑	↓
	Cht2	↓	↓	↓	↓	↓	↓	↓	↓
	Cht3	↓	×	↓	↓	↓	×	↑	↓
	Cht5	↓	↓	↓	×	↓	↓	↓	↓
	Cht6	↓	↑	↓	↓	↑	×	↑	↑
	Cht7	↓	↓	↓	↑	↓	↑	↓	×
	Cht8	↓	↓	↓	↓	↓	↑	↓	↓
	Cht10	↓	↓	×	↓	×	↑	↓	↑
	IDGF	×	↑	×	↑	↓	×	×	×
	ENGase	↓	↓	↓	×	↓	↓	×	↓

Supplementary Table 3. The down/up-regulation of genes related to chitin metabolism by knockdown of *ApTRE* and *ApTPS* expression with RNAi.. ↓, down-regulation mRNA expression; ×, no difference; ↑, up-regulation mRNA expression.

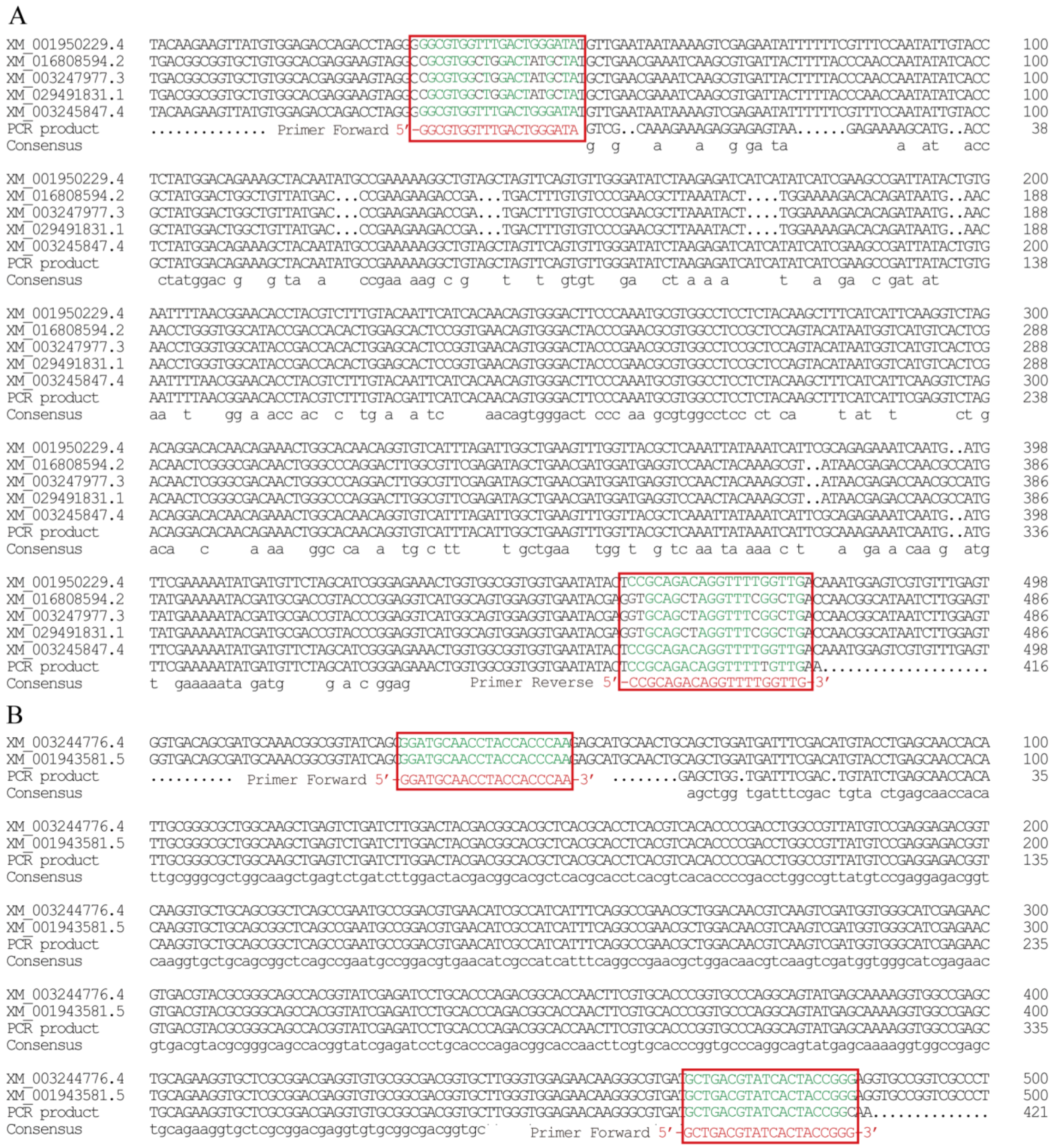

Supplementary Figure 1. The alignment of *ApTRE* (A) and *ApTPS* (B) isomers with PCR primers and PCR products. (A) Five *ApTRE* isomers (XM_001950229.4; XM_003245847.4; XM_016808594.2; XM_003247977.3 and XM_029491831.1) and (B) two *ApTPS* isomers (XM_003244776.4 and XM_001943581.5).

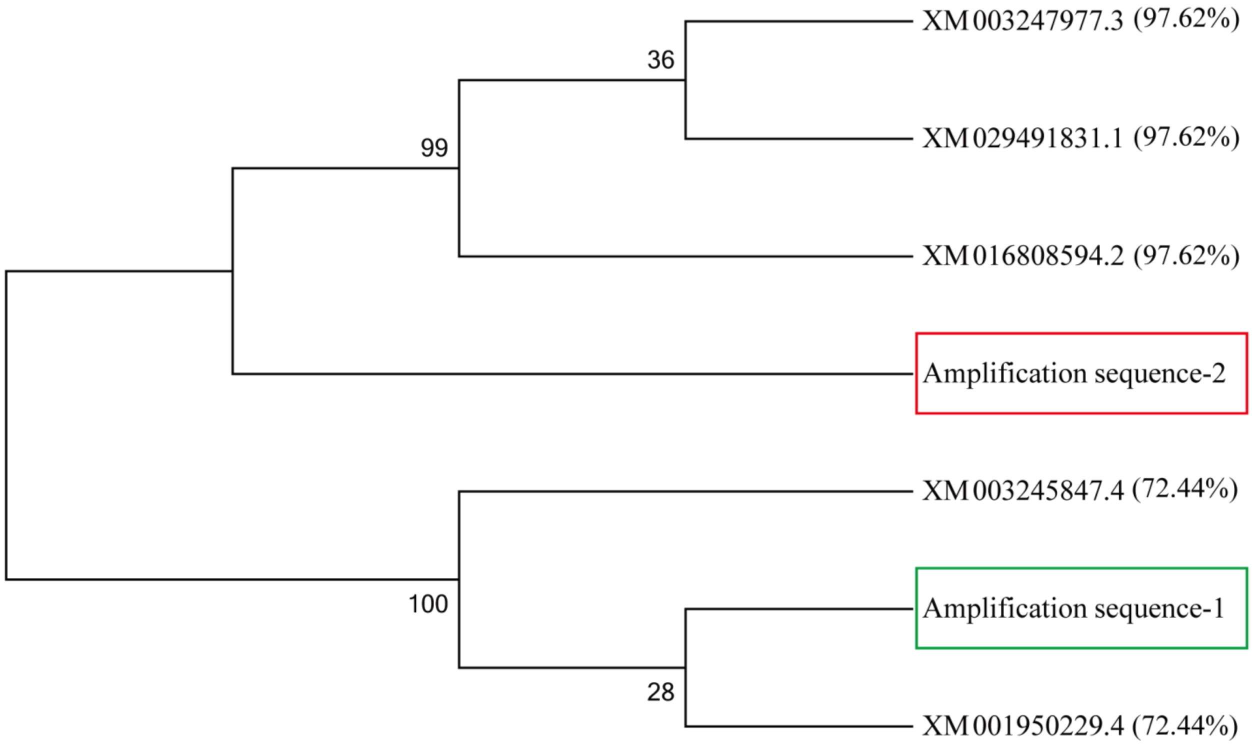

Supplementary Figure 2. Phylogenetic tree of *ApTRE*. The tree generated through ClustalW alignment using MEGA 7.0. The bootstrap values, expressed as percentages of 1000 replications, are shown at branch points. Amplification sequence-1 of *ApTRE* is highlighted with a green box and amplification sequence-2 of *ApTRE* is highlighted with a red box. The sequences were obtained from GenBank under the accession numbers: XM_016808594.2, XM_003247977.3, XM_029491831.1, XM_001950229.4 and XM_003245847.4. The *ApTRE* amplification sequence-2 by *ApTRE* primer：
CTTAAATACTTGGAAAAGACGCAGAGTGCAATTTTAACAGAACACTTACCTCTTTGAAGA
CTCATCCCAACTCTGTTTGTTCCCACTGCGCCGCCGCCTCCGCTCCAGTACATAATGGTAT
AAGGACGCCCCAACTGGGCGACAACTGGGCCCAGGTTTTTTTTAGATTGGTGGAAAATG
GGTTACCCTCCACTTATATACTTTTTGCACAGAAATTGGGATGTTCCCTGCTATGATGTTCT
AGCATCGGTAGATTTTGCTGGCATGGAGGTGAATACGAGGTGCAGCTAGGTTTCGGCTG.

>XM_001943581.4 PREDICTED: *Acyrthosiphon pisum* alpha,alpha-trehalose-phosphate synthase [UDP-forming] (LOC100165203), transcript variant X1, mRNA

AGCTGCAGTGGCGTTACAACAAATAATTACGATCATAGATCCGCGAACGAACTCATCGAACGTTATATTATAATATTATTTTTATTGCCATTCACGCGA

CCAATGTGCTTTAATAATATCATAAATCCGTATCGAACTCGTATGTGGTGTAATACTGTGCAATAATAATGCCTATAAACGAGTGAATCTGATAACACA

ATAAAATATGATAACACAAAATGGTAATGATCGAATTTTCCCAACCCACGCAGCATAATACGTTAATCGCGCGTACGGTGTGTGCGTATTATAATATTA

AGTTGTCTTGGTTCCGATTTACGATAAGGACGCGTTTTATTTAATATATATAACCGGAGCCGTACAACAGACAGATAGTCTACAGTCAACACTCGAAC

GTTCGCATTGTCTCAACTGCAGTCTGATCAATCAATCGATATAACAATACAACATACGTAATAGAAATATAATAATAATATCATCTCAACAATCGCGTC

ACCATCATGGCCAGCCGCCGTGTAAGCATCAATGATTACGACTCCAATGCGCCCAGTAACTTATTCCGGACCAACGGTCAAAATTTATCCGGCATGG

GAAACAAAAAAAGAGAGATCAACGCGCCTCTCATCGTAGTTTCCAATCGACTTCCGTTTGTTTTGGGTAAAAATGCAGACGGAGAATTAGTGCGG

AAACAAAGCGCGGGTGGTCTTGTAACGGCCGTCGCTCCAGTTGTTGTCGAATGTAAAGGCACATGGATTGGATGGTCTGGTTCGTTCGATTTAAAA

CCCAATGATCCAATTCCGGAATCCGATCCGGAAGACAAAGCTCCTACCGCTGGACTCAAATCTAGTCAAATTATTCCGGTACACTTAGATAAAACGG

AAATTGAAGAATTCTACAATGGTTGTTGTAATGGCACGTTTTGGCCCTTATTTCACTCGATGCCCACCGACCGTGCCAATTTCGTTTCTAGGGACTGG
RT-qPCR Primer Forward 5’-ACCGACCGTGCCAATTTCGTTT-3’
AAAACGTACTGCAAAGTAAACGACAAGTTTGCCGATTGCACGCTGGACGCCCTGCGGGATGCTATAAAATCCCTAGACGACGCTGGAGATCACCA
RT-qPCR Primer Reverse 5’-CG
AACAGCCCCCTTGGTTTCGATTCATGATTATCAGTTACTGACGACCGCGACCAAAATCCGTCAGGTGTGCGAAGCCGAAAACCTCAGGTGTAAGCT
AAACCAAGGGGGCTGTTTG-3’
GGGATTCTTCTTGCACATACCGTTCCCGTCGTGGGACATTATGAGGTTGTTGCCTTGGGACGACCAAGTTCTCCAGGGAATGTTGGCTTACGATTTC

ACCGCCTTCCACATCGAGGACTATTGCTTGAATTTTATTGACTGCTGCTGTCGGCGGCTTGGGTGCCGCGTGGACCGGACCAACATGCTGGTGGAG

CTGGCCGGACGGACCATACAAGTGAAGGCGCTGCCGATTGGCATACCATTTGACCGTTTCGTGCAACTATCGGAGAAGGCGCCGGCGTTCCTAAAC

ATGTCAGAAGATGTCAAGGTGATACTGGGCGTGGACAGGCTGGACTACACCAAGGGTCTGGTGCACCGAATCCTGGCGTTCGAGAAATTCCTGGA

CAAGTACCCGGAGCACCGGGAAAAAGTGGTGCTGCTGCAAATCTCGGTGCCATCCAGGACGGACGTCAAGGAGTACAAGCAACTCAAGGACGAG

ACCGAGCTGCTGATCGGCCGCATCAACGGAAGGTTCTCGATGCCCAACTGGTCACCGATCCGTTACATCTACGGGTGCCTGAGCCAGGAGCAGCTG

GCCGCCCTGTACCGAGACTGCGCGGTGGCATTGGTGACGCCGCTCAGGGACGGCATGAACCTGGTTGCCAAGGAGTTCGTGGCGTGCCAGATCCG

GACGCCGGGTGTGCTGATCCTGTCACCATTCGCCGGCGCCGGTGGCACCATGCATGAGGCGCTACTGGTCAACCCATACGAGCTGGACGAGATGGC

CAACGTGCTGCATCGAGCCCTGCGGATGCCGCTTGACGAGCGTGAACTGCGCATGACACAGCTCAGGCACCGAGAACAGCTGCTGGACGTCAACT

ACTGGATGACGTCGTTCTTCTCGTCGATGGGCGCGCTCAGCGACGGTGGCGGTGACAGCGATGCAAACGGCGGTATCAGCGGATGCAACCTACCA
dsRNA PCR Primer Forward 5’-GGATGCAACCTACCA
CCCAAGAGCATGCAACTGCAGCTGGATGATTTCGACATGTACCTGAGCAACCACATTGCGGGCGCTGGCAAGCTGAGTCTGATCTTGGACTACGAC
CCCAA-3’
GGCACGCTCACGCACCTCACGTCACACCCCGACCTGGCCGTTATGTCCGAGGAGACGGTCAAGGTGCTGCAGCGGCTCAGCCGAATGCCGGACGT

GAACATCGCCATCATTTCAGGCCGAACGCTGGACAACGTCAAGTCGATGGTGGGCATCGAGAACGTGACGTACGCGGGCAGCCACGGTATCGAGA

TCCTGCACCCAGACGGCACCAACTTCGTGCACCCGGTGCCCAGGCAGTATGAGCAAAAGGTGGCCGAGCTGCAGAAGGTGCTCGCGGACGAGGT

GTGCGGCGACGGTGCTTGGGTGGAGAACAAGGGCGTGATGCTGACGTATCACTACCGGGAGGTGCCGGTCGCCCTACGCGAACACCTGGAGCGC
dsRNA PCR Primer Reverse 5’-CCCGGTAGTGATACGTCAGCT-3’
CGAGCGGTCGAGATATTTGAGGCAGCCGGGTTCGAGCCGCACCGTGGGTTCCTGGCCATCGAGGGCAAGCCACCCGTTACGTGGGACCAGGGCCG

CGCGTCCATTTACATACTGCGCACCACATACGGAGTCGACTGGTCCGAGCGGGTGCGTGTCGTGTACGCTGGCAACGAGGACGCCATGCTGTCGCT

CCAGGGCATAGCATGCACGTTCCGCGTCGACCCATCGCCGATGGTCCAGACGGCCGCAGACTTCCGGCTGTCCGGTCCCGACGCCGTGCTCACCAT

GCTCAAGTGGGTGGAGAAGGAGATGCACAAACGGCTGCCGACGCTCAGCGGCCGGACGGTCAGCAATAGCCGGGTGCAGAACGCTGCGCTGGC

GCACAGCATCCGGTCGCAGATGAGCATCGACCAGAAGGAAGAGGAAAAGGAGAAGATGATCCTGCTGGCCCGGAGGTCTAAGCTCACCACCACT

TGAGCGGCCGTTCGAAAGGATTAGTAAGACTAAGCACGCAACACGCCGCGTTACGACATTTTAATTGATGTATCATAACTGTACTCGACGGCATGAC

Supplementary Figure 3. The positions of RT-qPCR and dsRNA primers for *ApTPS*. The green and red sequences were target sequences and primers sequences, respectively. The blue rectangular box indicated RT-qPCR primer and target sequences, and the black rectangular box indicated dsRNA primer and target sequences.

>XM_003245847.3 PREDICTED: *Acyrthosiphon pisum* trehalase (LOC100161043), transcript variant X1, mRNA

TGGGTCTTGCGTTCCGATAAAGGCGCATTGTACGCTGGTCATTAGTGCAGTCGATTCGTGCAAGGCCTGTGGGAAACCTGTGAAGGAGTCTCAGGG

TCCAACTTAAAAATCTCACTTTTTTGTTTTTGTTTTTTAATATTCAAATTTTTAATTGAAAAAAAAATAAATATTTTACTTCAAAATAGATTATTATACA

GATTATGCTAGTTTATTGGAAGAAATAAAATTAAAATATTATTTTTCTCTCTGTGTACACAATATCCGCGAATTATTGTAATTCGTTCAATATTTTTGAA

ACTATGCGTATTACTAACTTACTGGTCGTGTGTCTGGCACATTTTGCGTATTACACACATGCAAATAATCAGGAATTTGTTCATTTGGCAAGAGGATAT

TATCACGTATCCAATGGACTTCAGGCATCGTGTCAAAGCCAAATATACTGTGAAAGTGATCTACTGAAGGACGTACAACTAGCTCACATTTTTCCAG

ATTCGAAAACTTTTGTGGATATGAAACTAAAATACTCAGAATCTGAGATTTTAAAAAACTACCAAGTATTGAAAGACGGTAACAACGGTGTAGTGCC

RT-qPCR Primer Forward 5’-AACAACGGTGTAGTGCC

CAAAGAAAAGATAGTTAAATTTGTCGACGAACATTTCATGGACGGTGATGAGTTAGAGGTATGGACCCCTTCCGACTTTAATGAATCTCCTTCGATC

CAA

GCCAATCGGATTAAAGACAAGAATTACAAACAGTGGGCATTGGGATTAAACCAAGTATGGAAAACCCTAGCGAGGAAAGTTAAGGACGACGTGAG

RT-qPCR Primer Reverse 5’-TCCCAATGCCCACTGTTTGT

ATTACATCCTGACAGATATTCGCTGATATGGGTTCCAAATGGTTTTGCCATACCCGGAGGCCGATTTAGAGAACTTTACTATTGGGACACGTACTGGA

TTGTTAACGGGATGTTATTGTGTGACATGTCGACAACAGCTAGAGGTGTTATTGACAATATATTATCGTTGGTGCTGCAATTTGGTTTCATGCCGAAT

GGTGGACGAGTGTACTACCTGAATAGATCACAACCGCCAATGGTGACGTTGATGGTATCAAGCTACTATAAAGCAACCAACGACTTTGAATATGTAA

AAAAAGTCATATCTATCTTAGATAGTGAATTCGAGTTTTGGACGGAAAACCGAATGGTTACTTTCGAGAAGAACGGCAAATCGTACACAATGGCAA

GATACTACGCACCGTCAAGGGGTCCAAGACCAGAGTCTTATAGAGAGGACTACGAATCAGCAGAATTTTTGAAAACTGAAAACGAAAAACAAGAA

TTGTATACCCAAATTAAGTCTGCGGCAGAGACCGGATGGGACTTTTCAAGTAGATGGTTTATAACTGCCAACGGCTCGGATCGTGGGATATTGGCTG

ATATAAAAACGACGTATATAATACCAGTGGATTTGAACTGTATATTGCACAAGAATGCACTGTTGTTGAGTTCGTGGTATAGTAAGATGGGAGATACA

ACTAAAGCCGAGAAATATCGTGCGATCGCTGAGAAACTCGTCTACAGTATACAAGAAGTTATGTGGAGACCAGACCTAGGGGCGTGGTTTGACTGG

dsRNA PCR Primer Forward 5’-GGCGTGGTTTGACTGG

GATATGTTGAATAATAAAAGTCGAGAATATTTTTTCGTTTCCAATATTGTACCTCTATGGACAGAAAGCTACAATATGCCGAAAAAGGCTGTAGCTAG

GATA

TTCAGTGTTGGGATATCTAAGAGATCATCATATCATCGAAGCCGATTATACTGTGAATTTTAACGGAACACCTACGTCTTTGTACAATTCATCACAACA

GTGGGACTTCCCAAATGCGTGGCCTCCTCTACAAGCTTTCATCATTCAAGGTCTAGACAGGACACAACAGAAACTGGCACAACAGGTGTCATTTAG

ATTGGCTGAAGTTTGGTTACGCTCAAATTATAAATCATTCGCAGAGAAATCAATGATGTTCGAAAAATATGATGTTCTAGCATCGGGAGAAACTGGT

GGCGGTGGTGAATATACTCCGCAGACAGGTTTTGGTTGGACAAATGGAGTCGTGTTTGAGTTTTTAAATAGATGGGGCGATACATTATCTAACGGGA

dsRNA PCR Primer Reverse 5’-CAACCAAAACCTGTCTGCGG-3’

TAAACGGATGTACTACCGGTTATAGATATTTGTGTGATTTCAATAGGATCAGACAACAGTTAATGTTTTGGACTTGAGAAGACACGGGTAATCGTCTT

ATTCGCCTATGCACCGGAAACTGATTATACTTAGGTACCTAATTAAATTTACCATGCGTCAAACGGCTGAAACGCTGATAAATTTTATTCAAAGAAGA

AACTCAGTATTAATTAATTATTACATAATAATTTTTATTATCTAAAAAATATTATTTTTAGATAATTTTTATTAAACAAAAGACAACACATACCTAGAATT

ATTGATTTTTTATACACACAAAAACAATGCAACGCGAAATAACATATGTTTATTGATAAATAATTTGTTAAAATATTGTTTCATTTATATACATTGTATAA

GATAGTTTTACACTTGTTTTAATTACTATAAGGAATTGTTGTTGATGTTAATATTATTAAAAAAAGAACAGGCAATTTTTAATAGTTAAAATACTTGTAA

TTTATATATTATACACTTTTTTGCATGTACAACAGGTATTTGATATGCCTTATTAATTTATTGGAAATATATAAAAAAAAATTGAAAAACAA

Supplementary Figure 4. The positions of RT-qPCR and dsRNA primers for *ApTRE*. The green and red sequences were target sequences and primers sequences, respectively. The blue rectangular box indicated RT-qPCR primer and target sequences, and the black rectangular box indicated dsRNA primer and target sequences.

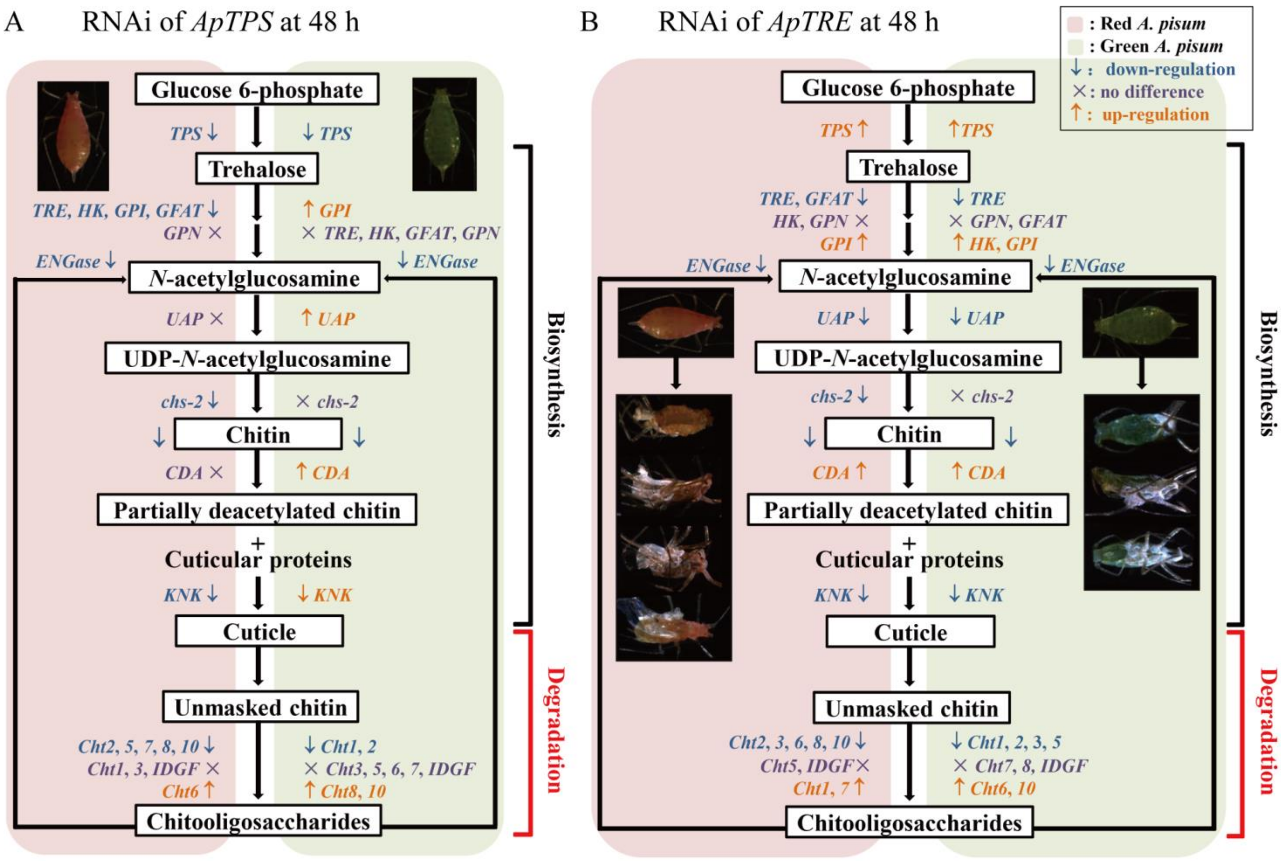

Supplementary Figure 5. Key enzymes and genes involved in chitin biosynthesis and degradation in *A. pisum*. (A) Effect of RNAi of *ApTPS* on chitin metabolic pathway at 48 h. (B) Effect of RNAi of *ApTRE* on chitin metabolic pathway at 48 h. The colour of pink represents the red *A. pisum*, the colour of greenish represents the green *A. pisum*, the blue ↓ and letters represent down-regulation mRNA expression, the purple × and letters represent no difference, the orange ↑ and letters represents up-regulation mRNA expression.
